# Factors Associated with Urinary Incontinence in Female Weightlifters

**DOI:** 10.3390/healthcare14030381

**Published:** 2026-02-03

**Authors:** Sofia Lopes, Manon Becam, Carla Pierrot, Julie Réard, Alice Carvalhais, Ágata Vieira, Gabriela Brochado

**Affiliations:** 1 Departamento de Tecnologias de Diagnóstico e Terapêutica, Escola Superior de Tecnologias da Saúde do Tâmega e Sousa, Instituto Politécnico de Saúde do Norte (IPSN), 4585-116 Gandra, Portugal; man79bec@gmail.com (M.B.); pierrotcarla@gmail.com (C.P.); julie.reard@gmail.com (J.R.); alice.carvalhais@ipsn.cespu.pt (A.C.); agata.vieira@ipsn.cespu.pt (Á.V.); gabriela.brochado@ipsn.cespu.pt (G.B.); 2 ESS, Escola Superior de Saúde, Instituto Politécnico do Porto, Rua Dr. António Bernardino de Almeida, 400, 4200-072 Porto, Portugal; 3 H2M—Unidade de Investigação em Saúde e Movimento Humano, Instituto Politécnico de Saúde do Norte, CESPU, CRL, 4760-409 Vila Nova de Famalicão, Portugal; 4 Centro de Investigação em Reabilitação (CIR), Escola Superior de Saúde, Instituto Politécnico do Porto, Rua Dr. António Bernardino de Almeida, 400, 4200-072 Porto, Portugal; 5 Laboratório Associado de Energia, Transportes e Aeronáutica, Instituto de Ciência e Inovação em Engenharia Mecânica e Engenharia Industrial, Faculdade de Engenharia, Universidade Porto, 4200-465 Porto, Portugal; 6 Departamento de Fisioterapia, Escola Superior Saúde Santa Maria, Trav. Antero de Quental 173/175, 4049-024 Porto, Portugal

**Keywords:** female athletes, pelvic floor disorders, strength training

## Abstract

**Background/Objectives**: Urinary incontinence (UI) is common among women practicing sports, particularly those involving heavy lifting or high-impact movements that increase intra-abdominal pressure. UI can negatively affect social life, self-confidence, and motivation to remain active. This study aimed to examine the associations of sociodemographic, training-related, obstetric, and surgical factors with UI in female weightlifters. **Methods**: This cross-sectional study included 84 French women who regularly practiced weightlifting. Participants completed a structured questionnaire collecting sociodemographic and gynecological information, as well as the Urinary Symptom Profile (USP). Data were analyzed using appropriate inferential statistical tests, including the Mann–Whitney U test, Student’s *t*-test, chi-square test, and Fisher’s exact test, as applicable. A 95% confidence level was adopted for all analyses. **Results**: Among participants (aged 15–49 years), 51 (60.7%) reported involuntary urine leakage, and 31 (36.9%) scored 1–3 on the USP stress incontinence subscale. Most participants were non-smokers (73.8%), with a median of 3.5 years of weightlifting experience, four weekly training sessions, and six–seven competitions per year. No significant associations were found between UI and sociodemographic factors, obstetric history, previous surgeries, or training characteristics. Maximal lifts in Clean & Jerk and Snatch exercises were also similar between participants with and without UI. Slight trends suggested a higher UI prevalence among women with vaginal deliveries, episiotomies, or vaginal lacerations. Regarding athletes with and without UI, no differences were found (*p* > 0.05) with respect to weightlifting belt use or the breathing phase during load lifting. **Conclusions**: UI is common among female weightlifters, but in this study, was not associated with sociodemographic factors or weightlifting practices. These findings indicate that UI prevalence cannot be explained by the variables studied and highlight the need for further research into other potential contributing factors.

## 1. Introduction

Urinary incontinence (UI) is defined as the involuntary leakage of urine [1]. This clinical condition refers to a dysfunction of the pelvic floor (PF), affecting the anatomical and functional integrity of the PF, which includes the supporting muscles and connective tissues that control the pelvis and its organs [1,2]. It can manifest as weakness, leading to issues such as stress and fecal incontinence, pelvic organ prolapses, or, conversely, as hypertonic disorders characterized by dysfunctional muscle activity [1,2]. Conditions such as urinary urgency, pelvic pain, and other voiding dysfunctions can arise as a secondary effect of other pelvic pain disorders [3].

This condition affects both women and men but is more common in females. The most common cause of UI in men is prostate enlargement or changes resulting from treatment for prostate cancer [4]. The different stages of a woman’s life, such as pregnancy, childbirth or menopause, can cause bladder or pelvic floor muscle (PFM) dysfunction, resulting in UI of any type [5]. There are three main types of UI: stress incontinence, urge incontinence, and mixed incontinence [6]. One function of the PF is to regulate bladder and bowel storage and emptying, signaling when elimination is necessary by coordinating the opening and closing of the urethral and anal openings to help maintain continence [7]. When functioning normally, the PF has three main roles: to support the pelvic viscera against changes in intra-abdominal pressure (IAP), to act as a constrictor to maintain urinary and fecal continence [8,9,10] and enable successful sexual function [7]. From an anatomical point of view, the PF consists of muscles, ligaments and fascia that support the bladder, reproductive organs and rectum. These soft tissues are surrounded by the bony structure of the pelvis, which consists of four bones: two hip bones, the sacrum, and the coccyx [11].

The average prevalence of UI is 30% among middle-aged women, and it can affect 47% of women who exercise regularly [12]. Regarding this condition in sport, scientific literature has already demonstrated that UI is related to the intensity of impacts during training. The prevalence of UI ranges from 5% in low-impact activities to 80% in high-impact activities, such as trampolining [13]. In addition to the intensity of the impacts, the amount of training influences UI symptoms, with a prevalence of incontinence ranging from 10 to 80% [13]. According to Jacome et al. [14], 41% of female athletes who regularly lift weights also have UI. Weightlifting is a strength and power sport consisting of two movements: the Snatch and the Clean and Jerk [15]. These combine lifting heavy loads and the Valsalva maneuver (forced exhalation against a closed glottis) [16], which significantly increase the pressure on the PF [17]. Weightlifting causes a significant increase in IAP, especially with the use of the Valsalva maneuver, which is often used to stabilize the torso when lifting heavy loads. The increase in IAP is transmitted to adjacent pelvic structures, including the urinary bladder and the bladder neck region [18]. Generally, the fear of having urinary problems, especially during public competitions, limits the intensity of effort, leading some athletes to abandon the sport [17]. Many women avoid certain exercises (e.g., jumping, heavy weights) or adapt their training, which can compromise their training progress and overall health. Ashton-Miller and DeLancey [19] showed that 20% of women with UI restrict their physical activities, which can impact the quality of life of athletes by reducing their social life, daily activities, and psychological state [20], and can result in decreased physical condition, weight gain, and even trigger symptoms of depression [19,21].

Physiotherapy plays an essential role in the rehabilitation of this clinical condition (Ghaderi and Oskouei [22]). According to Wallace et al. [23], pelvic physiotherapy has solid evidence-based support and clear benefits as a first-line treatment for most PF disorders. As UI is recognized as a common problem among athletes [24], individualized interventions and training plans are increasingly recommended [22]. Although the study was initially planned to include participants from both France and Portugal, the prevalence of regular weightlifting practice differs substantially between these countries. Therefore, the study was conducted solely among French female weightlifters to ensure adequate sample size and representative participation. This study aimed to examine the association of sociodemographic, training-related, obstetric, and surgical factors with UI among French female weightlifters.

## 2. Materials and Methods

A cross-sectional observational study was conducted. The target population of the study consisted of women who practice weightlifting in France. The selection was made for convenience based on a non-probabilistic sample. The following criteria were considered for sample selection: (i) women aged between 15 and 50 years [25]; (ii) regularly practiced weightlifting, defined as a minimum of two training sessions per week for at least 6 months [26,27]; (iii) provided informed consent to participate in the study. Women with cardiovascular disease, diabetes, and asthma [28], a history of neurological conditions affecting UI, moderate to severe overactive bladder, and moderate to severe dysuria [29] were excluded. This study was approved by the Ethics Committee of the Northern Polytechnic Institute of Health (IPSN) on April 2025 (58/CE-IPSN/2025). All participants provided written informed consent prior to participation.

The study focused on UI in female weightlifters, with data collected on the presence or absence of urinary leakage specifically during weightlifting training sessions. Leakage occurring during other daytime activities, such as laughing, running, or walking, was not assessed.

A self-administered questionnaire, developed based on previous studies on UI in athletes and weightlifters, was used to collect data. The questionnaire covered three domains: (i) sociodemographic information, including general characteristics, lifestyle habits, and medical history; (ii) weightlifting practice; and (iii) associated risk factors, including medical follow-up and obstetric history. In addition, participants completed the Urinary Symptom Profile (USP), which has been validated for the French population (Supplementary Materials). This comprehensive approach allowed for a detailed characterization of the study population and the variables relevant to UI [30]. The USP quantifies stress urinary incontinence (SUI) severity and provides sub scores for overactive bladder (storage symptoms) and bladder emptying symptoms. This assessment enabled the exclusion of participants who had excessively high scores in the overactive bladder and/or dysuria domains, corresponding to moderate or severe manifestations of these conditions. Regarding the USP score, any score other than zero was considered indicative of lower urinary tract dysfunction [29].

### 2.1. Procedures

#### Recruitment

Participants were recruited through French weightlifting clubs that provided signed authorization to participate in the study. The questionnaire link, together with a flyer containing a Quick Response (QR) code, was sent by email to these clubs. While data on the total number of clubs and the number of female weightlifters in France are not publicly available, the study included all women from participating clubs who met the inclusion criteria.

### 2.2. Statistical Procedures

Data collection took place between 8 April and 15 May 2025. After this period, the data was exported to Excel and transferred to SPSS v30 software. To examine the relationship between risk factors and the presence of UI in the study sample, the symmetry of the scalar variables was assessed. Symmetric variables included age, number of competitions per year, Snatch one-repetition maximum (1RM), and number of pregnancies. Asymmetric variables comprised body mass index (BMI), daily water intake, training experience in weightlifting, weekly training frequency, and Clean and Jerk 1RM. Based on these results, either the student’s *t*-test or the Mann–Whitney test was applied to compare variables between groups with and without urinary leakage. The chi-square test or Fisher’s exact test was used to assess associations between qualitative variables.

## 3. Results

**Figure 1:**
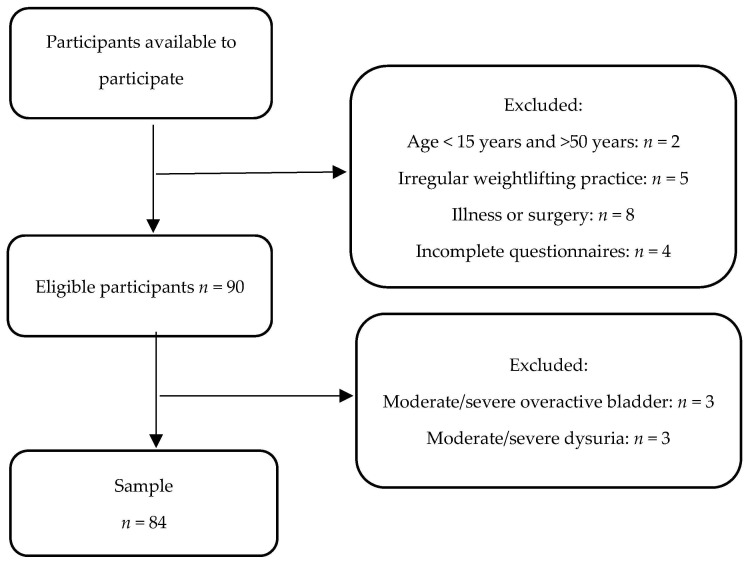
Flow diagram of the study sample.

Analysis of the questionnaire responses allowed us to verify the eligibility of participants for the study. Of the 109 women available to participate, 26 were excluded, yielding a final sample of 84 (Figure 1).

Table 1 shows the sociodemographic profile of the athletes, as well as the variables associated with sports practice.

The sample consisted of 84 athletes aged between 15 and 49 years. Most began weightlifting 2 to 6 years ago (56; 66.67%), with a median (P25–P75) training frequency of 4 (3–5) times per week. On average, they participate in 6.5 (4.0–8.0) competitions per year. Regarding smoking habits, most athletes (62 (73.8%)) were non-smokers. Among smokers, median consumption was 49 (28–70) cigarettes per week, with a median smoking duration of 7 (5–15) years. Among the participants, 51 (60.7%) reported experiencing involuntary urinary leakage.

Table 2 shows the different risk factors for UI in the sample.

Higher percentages were observed in the UI group for older age, higher BMI, and prior vaginal delivery, episiotomy, or vaginal lacerations. However, none of these differences have reached statistical significance.

**Figure 2:**
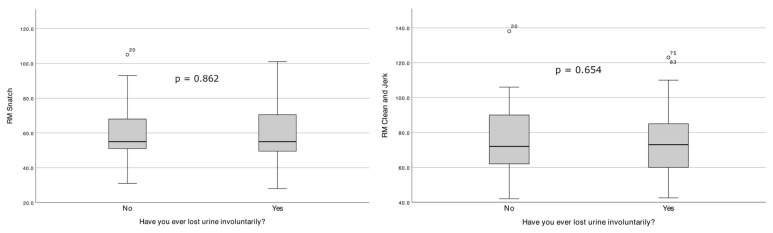
Distribution of 1RM Snatch and 1RM Clean & Jerk according to the presence or absence of involuntary urine leakage.

Figure 2 shows the distribution of 1RM Clean and Jerk and 1RM Snatch according to the presence or absence of involuntary urinary leakage.

Regarding athletes with and without UI, no differences were found (*p* > 0.05) with respect to weightlifting belt use or the breathing phase during load lifting.

## 4. Discussion

The present study examined the influence of sociodemographic and obstetric factors on UI and evaluated whether weightlifting practices are associated with UI among female weightlifters. In our sample, the prevalence of UI affects more than half of female weightlifters. Although relevant, this estimate is higher than reported previously, which indicates that less than half of female weightlifters experience UI [31]. The study by Wikander, Kirshbaum, Waheed, and Gahreman [31] reported a slightly lower prevalence of UI compared with the present study in a cross-sectional, survey-based sample of 191 competitive female weightlifters. In their study, UI frequency and severity were assessed using the Incontinence Severity Index, rather than a binary presence/absence definition, which may partly account for the differences in prevalence observed between studies. Regarding the influence of the different studied variables, no differences were observed in relation to involuntary urinary loss in the study population.

These findings indicate a potential association; however, they should be interpreted with caution due to the design and sample size. In line with this pattern, Huebner et al. [32] noted that multiple factors may contribute to the age-related differences in UI prevalence among master female weightlifters.

Differences in participants’ age ranges can significantly influence outcomes, as older athletes may have a different health status and training history compared to younger ones. For example, the referred study reveals a higher prevalence of moderate or severe UI in weightlifters than in the general population, which suggests that the athletic environment may introduce unique risk factors. Additionally, prior participation in high-impact sports appears to increase the risk of developing UI among these athletes. Concurrently, mental health factors like depressive mood have been identified as risk factors for UI, adding another layer of complexity to how age might interact with both physical and psychological characteristics in this context. Variability in training regime and sport history could further account for the discrepancies across studies, indicating that multiple factors must be considered to understand the broader population characteristics impacting UI prevalence in master female weightlifters [32]. The results indicate a possible pattern that may warrant further investigation in future studies with the duration of sport practice, which may also influence urinary loss. Likewise, the use of a weightlifting belt may act as a potential aggravating factor for UI, as reported previously. Considering the conditions of delivery, vaginal delivery tends to be more traumatic for the PF and cause UI in the women involved, compared to Cesarean delivery. Episiotomy and vaginal lacerations appear to be risk factors for UI. These results seem to be consistent with the data currently available in the scientific literature. The studies reviewed in the systematic analysis developed by Wang et al. [33] indicate that episiotomy does not generally provide a protective effect against UI. In fact, only one of the two studies that specifically analyzed urge UI found an association with episiotomy, suggesting a lack of consistent evidence supporting episiotomy as a risk factor for UI. Additionally, across three register linkage studies involving 37,849 patients, the findings were inconsistent [31]. One study indicated an increased risk of subsequent anti-incontinence surgery following episiotomy, while the other two found no significant associations between episiotomy and rates of anti-incontinence procedures. Overall, the evidence points toward a complex relationship between episiotomy and UI, lacking definitive conclusions about risk factors [33]. Regarding other factors, this study suggests a possible pattern that may warrant further investigation in future studies whereby higher BMI values are associated with a higher probability of UI. These results are not consistent with those obtained by Wikander, Kirshbaum, Waheed, and Gahreman [20], who did not observe any influence of BMI on the presence of UI in weightlifting athletes. This discrepancy may be due to differences in sample characteristics.

From the perspective of weightlifting practice, the number of competitions held per year, the maximum weight lifted in the two exercises, and the athlete’s years of sporting practice were not associated with UI. These results have already been found in the literature, namely in the study by Wikander, Kirshbaum, Waheed, and Gahreman [20], where they also did not find that the number of years of sporting practice was a factor in the development of UI. These authors also state that the 1RM of the Snatch and Clean and Jerk has no impact on the condition studied. However, this study did not reveal an influence between UI and, respectively, the use of a weightlifting belt, the number of training sessions per week, and the breathing phase in the stage in which the movement is performed [20,32,34]. The sport characteristics of the sample produce differences in outcomes. The findings indicate that maximum effort lifts, such as Snatch and Clean and Jerk during competition, were less likely to result in UI compared to maximum effort lifts performed in training. While heavy loads and high repetition sets were key factors that provoked UI, particularly with squats, there is no significant evidence connecting these specific weightlifting techniques (Snatch and Clean and Jerk) directly with increased occurrences of UI. Overall, women reported more instances of leakage during high repetition sets and heavy lifting, but maximum competition lifts were less impactful on urinary leakage compared to training [20]. Training sessions often include a substantial cardiovascular component (e.g., running and skipping rope), which may be more likely to provoke UI in women than weightlifting itself. Furthermore, many participants reported being aware of their fluid intake prior to lifting, with some intentionally restricting fluid consumption before training or, in some cases, training while dehydrated [18,35,36].

Smoking was included as a variable given its established association with UI. Tobacco use has been linked to chronic cough and recurrent increases in IAP, alterations in connective tissue quality, and bladder irritation, all of which may contribute to pelvic floor dysfunction and the development of UI [37]. Therefore, smoking status was considered relevant for contextualizing the study population and interpreting the findings.

### Limitations

Although this study is based on an evidence-based approach, supported by epidemiological and clinical data from national and international scientific literature, some inherent limitations are acknowledged. A limitation of this study is that, although stress, urge and mixed UI were distinguished conceptually in the introduction, the analyses did not differentiate between these subtypes. Consequently, the results reflect UI as a single construct, and subtype-specific conclusions cannot be drawn. The sample used in this study, comprising only 84 weightlifters, limits the generalization to the general population. A further limitation of this study is the potential presence of selection bias, which may have influenced the estimated occurrence of UI. Depending on participant characteristics and self-selection mechanisms, this bias could have led to either an underestimation or an overestimation of UI prevalence. Therefore, the findings should be interpreted with caution. Furthermore, the self-completion nature of the questionnaire may lead to an underestimation of urinary symptoms, which could have been reduced by applying for a clinical test.

Additionally, UI was analyzed as a binary outcome (presence versus absence), which, while aligned with the study objectives, may contribute to an overestimation of prevalence and limits more nuanced interpretation based on symptom frequency or severity. Furthermore, participants’ responses may have been influenced by factors such as embarrassment, shyness, or the desire to project a positive image, leading them to understate their UI symptoms or overstate their sporting activity. Consequently, risk factors identified in the general population may not be fully applicable to this athletic population. For example, it is plausible that women who practice weightlifting show more effective recovery of PF function after childbirth, possibly associated with improved neuromuscular function and prior strengthening of these muscles.

Nevertheless, this study addresses a relatively unexplored topic. The distinction between personal factors and weightlifting practice characteristics provides an opportunity to develop complementary lines of research. Future studies with larger quantitative samples, as well as qualitative and longitudinal designs, could further clarify the epidemiology of UI in female weightlifters, alongside athletes’ knowledge, preventive behaviors, and management strategies. Increased awareness and targeted prevention may improve management of UI and support adherence to training programs designed to respect female physiology and anatomy. From a clinical perspective, these findings reinforce the importance of targeted rehabilitation strategies, particularly pelvic floor muscle training, as a key component in the prevention and management of UI in female weightlifters, contributing to safer training practices and long-term adherence to the sport and interventions in women with a history of vaginal delivery and episiotomy.

## 5. Conclusions

In this sample of female weightlifters, no statistically significant associations were identified between the examined risk factors, including weightlifting practice variables, and the presence of UI. Although higher BMI, a greater number of weekly training sessions, and a history of vaginal delivery, episiotomy, or vaginal lacerations were descriptively associated with a higher prevalence of UI, these observations did not reach statistical significance and should be interpreted with caution. Given the limited sample size, the potential for selection and reporting bias, the binary assessment of UI, and the absence of subtype-specific analyses, these findings should be regarded as exploratory. Further studies with larger samples and more comprehensive assessment strategies are required to clarify potential associations and to confirm these preliminary observations.

## Figures and Tables

**Table 1:** Sociodemographic and sport characteristics of the sample (*n* = 84).

	*n* (%)	Median [P25–P75]
Age (years)		27.0 [24.0–33.0]
Weight (kg)		64.0 [57.3–72.8]
Height (cm)		163.5 [158.5–169.0]
BMI (kg/m^2^)		24.2 [21.7–26.5]
Time spent weightlifting (years)		3.5 [2.0–6.0]
Number of training sessions per week		4.0 [3.0–5.0]
Number of competitions per year		6.5 [4.0–8.0]
**Weightlifting activity**		
1RM Clean and Jerk (kg)		72.5 [60.0–86.8]
1RM Snatch (kg)		55.0 [49.3–70.0]
**Obstetric data**		
Number of births	22 (26.2)	
Vaginal birth	19 (22.6)	
Cesarean birth	6 (7.1)	
Episiotomy	8 (36.4)	
Manual intervention	4 (31.8)	
Vaginal lacerations	11 (50.0)	
**Smoking habits**		
Non-smoker	62 (73.8)	
Smoker	9 (10.7)	
No. of cigarettes/week		49.0 [28.0–70.0]
Years of smoking		7.0 [5.0–15.0]
Ex-smoker	13 (15.5)	
No. of cigarettes/week		70.0 [28.0–102.0]
Years of smoking		7.0 [6.0–10.5]
Time since abstinence from tobacco (years)		5.0 [2.0–12.5]

*n*: absolute frequency; %: relative frequency; kg: kilogram; cm: centimeters; BMI: body mass index; 1RM: one repetition maximum.

**Table 2:** Comparison of participant and training related variables between women with and without UI.

	With UI (*n* = 51)	Without UI (*n* = 33)	*p*
	*n* (%)		
Age (years) (*n* = 84)			
15–24	15 (17.8)	11 (13.1)	0.143 ^a^
25–44	33 (39.3)	22 (26.2)	
45–50	3 (3.6)	0 (0.0)	
BMI (kg/m^2^) (*n* = 84)			
<18.5 (underweight)	1 (1.2)	2 (2.4)	0.086 ^b^
18.5–24.9 (normal weight)	30 (35.7)	23 (27.4)	
25–29.9 (pre-obesity)	15 (17.8)	5 (5.9)	
≥30 (obesity)	5 (6.0)	3 (3.6)	
Daily water consumption (L) (*n* = 84)			
<1.75	23 (27.4)	19 (22.6)	0.148 ^b^
1.75–2.5	17 (20.2)	9 (10.7)	
>2.5	11 (13.1)	5 (6.0)	
Smoking habits (*n* = 84)			
Non-smoker	38 (45.2)	24 (28.5)	0.945 ^c^
Ex-smoker	8 (9.5)	5 (6.0)	
Smoker	5 (6.0)	4 (4.8)	
Time in practice (years) (*n* = 84)			
<2	7 (8.3)	2 (2.4)	0.796 ^b^
2–5	24 (28.6)	21 (25.0)	
≥5	20 (23.8)	10 (11.9)	
No. of training sessions/week (*n* = 84)			
<3	8 (9.5)	5 (6.0)	0.167 ^b^
3–4	29 (34.5)	11 (13.1)	
≥5	14 (16.7)	17 (20.2)	
No. of competitions/year (*n* = 84)			
<5	14 (16.7)	7 (8.3)	0.128 ^a^
5–10	24 (28.6)	16 (19.0)	
≥10	13 (15.5)	10 (11.9)	
Use of a powerlifting belt (*n* = 44)	26 (59.0)	18 (41.0)	0.749 ^c^
Breathing phase during load lifting (*n* = 72)			
Expiration	16 (22.2)	13 (18.1)	0.381 ^c^
Inspiration	10 (13.9)	7 (9.7)	
Apnea	15 (20.8)	11 (15.3)	
Number of births (*n* = 22)			
1	7 (31.8)	3 (13.7)	0.810 ^a^
2	9 (41.0)	1 (4.5)	
3	1 (4.5)	0 (0.0)	
4	0 (0.0)	1 (4.5)	
Vaginal birth (*n* = 19)	15 (79.0)	4 (21.0)	0.953 ^a^
Cesarean birth (*n* = 6)	4 (66.7)	2 (33.3)	0.480 ^b^
Episiotomy (*n* = 8)	7 (87.5)	1 (12.5)	0.613 ^c^
Manual intervention (*n* = 4)	3 (75.0)	1 (25.0)	1.000 ^c^
Vaginal lacerations (*n* = 11)	10 (91.0)	1 (9.0)	0.311 ^c^
Pelvic surgery (*n* = 8)	6 (75.0)	2 (25.0)	0.471 ^d^
Abdominal surgery (*n* = 8)	5 (62.5)	3 (37.5)	1.000 ^d^

*n*: absolute frequency; %: relative frequency; *p*: *p*-value; BMI: body mass index; L: liters; No.: number; UI: urinary incontinence; ^a^: Student’s *t*-test; ^b^: Mann–Whitney test; ^c^: chi-square test; ^d^: Fisher’s exact test.

## Data Availability

The data presented in this study are available on request from the corresponding author, as the study is part of an ongoing research project.

## Supplementary Materials

The following supporting information can be downloaded at: https://www.mdpi.com/article/10.3390/healthcare14030381/s1, Questionnaire in female weightlifters.

## Abbreviations

UI	Urinary incontinence
USP	Urinary Symptom Profile
PF	Pelvic floor
PFM	Pelvic floor muscle
IAP	Intra-abdominal pressure
IPSN	Northern Polytechnic Institute of Health
SUI	Stress urinary incontinence
QR	Quick Response
1RM	One-repetition maximum
BMI	Body mass index
*n*	Absolute frequency
%	Relative frequency
kg	Kilogram
cm	Centimeters
