# Supplementary material for: Factors Associated with Urinary Incontinence in Female Weightlifters
Source: Healthcare (Basel). 2026 Feb 3;14(3):381. doi: 10.3390/healthcare14030381 (PMC12897852; doi:10.3390/healthcare14030381)
Supplement: Supplementary file 1 [file healthcare-14-00381-s001.zip › healthcare-4053505-supplementary.pdf]

Evaluation of risk factors for urinary incontinence in female Weightlifters

* Obrigatória

Informed consent

1. If you agree to participate in this study, please give your consent by ticking all the boxes below: *

Selecione 3 opções.

- [ ] I have read and understood the information about the study.
- [ ] I understand that I may withdraw from the study at any time.
- [ ] I declare that I have read the above information and consent to the processing of my personal data.

General information:

2. Age (in years): *

3. Weight (in kg): *

4. Height (in cm): *

Lifestyle habits:

5. How many liters of water do you drink per day? *

6. Do you smoke? *

- Yes
- No
- Former smoker

7. If you answered "Yes" to question 6, for how long?

8. If you answered "Yes" to question 6, how many cigarettes do you smoke per day?

9. If you answered "Former smoker" to question 6, how long has it been since you quit?

10. If you answered "Former smoker" to question 6, how long did you smoke?

11. If you answered "Former smoker" to question 6, how many cigarettes did you smoke per week?

Medical history:

12. Do you have a diagnosed disease/condition? *

- ◯ Yes
- ◯ No

13. If yes, please specify which one(s):

14. Have you ever had pelvic surgery? *

- ◯ Yes
- ◯ No

15. If yes, please specify which one(s):

16. Have you ever had abdominal surgery? *

- ◯ Yes
- ◯ No

17. If yes, please specify which one(s):

Weightlifting practice

18. How long have you been practicing weightlifting? *

19. How many training sessions do you do per week? *

20. What is the duration of your warm-up before starting weightlifting? *

21. Do you practice this sport competitively? *

- Yes
- No

22. If yes, how many competitions do you participate in per year?

23. Weightlifting involves two main movements: the "Clean and Jerk" and the "Snatch". Please indicate your 1RM (maximum weight lifted in one repetition) for each movement:
Clean and Jerk (in kg): *

24. Snatch (in kg): *

25. Do you use a weightlifting belt? *

- Yes
- No

26. Are there specific movements during which you feel an increase in abdominal pressure? *

- ◯ Yes
- ◯ No

27. If yes, please indicate during which exercise(s) this occurs:

[ ]

28. Do you practice any other strength-training exercises besides weightlifting? *

- ☐ No
- ☐ Deadlift
- ☐ Squat
- ☐ Plank (core exercise)
- ☐ Others

29. If you answered "Others" to the previous question, please specify the exercise(s): *

[ ]

Medical supervision:

30. Do you have regular gynecological follow-up? *

- Yes
- Noo

31. If yes, at what interval do you have these appointments?

Obstetric history

32. Have you ever been pregnant? *

- Yes
- No

33. If yes, how many times?

34. What was the type of delivery for each pregnancy?

	Pregnancy 1	Pregnancy 2	Pregnancy 3	Pregnancy 4	Pregnancy 5
Vaginal delivery	○	○	○	○	○
Cesarean section	○	○	○	○	○

35. Have you ever had an episiotomy?

- Yes
- No

36. Was the use of forceps required during delivery?

- Yes
- No

37. Was a manual intervention required

- Yes
- No

38. Did you experience vaginal tears during delivery?

- Yes
- No

Urinary incontinence symptoms and prevention

39. Have you ever experienced involuntary urine leakage? *

- ◯ Yes
- ◯ No

40. If yes, did this occur during a weightlifting training session?

- ◯ Yes
- ◯ No

41. Have you ever undergone a medical examination of the pelvic floor muscles? *

- ◯ Yes
- ◯ No

42. **Contracting the transverse abdominal muscle and the pelvic floor means activating the deep muscles that support posture and internal organs. The activation of these muscles should be gentle and controlled, without holding your breath. Transverse abdominal muscle:** Imagine you want to tighten your stomach as if to make your pants fit better, without holding your breath. You should feel a slight, deep tension around your navel, as if a band were wrapping around your torso. **Pelvic floor muscles:** It's like trying to stop the flow of urine or prevent passing gas. You should feel a slight lift inside the pelvic region, without contracting your glutes or thighs. **Knowing this, do you contract your pelvic floor muscles during exercise?** *

- ◯ Yes
- ◯ No

43. Knowing this, do you contract your transverse abdominal muscle during exercise? *

- ◯ Yes
- ◯ No

44. Have you ever undergone pelvic floor rehabilitation (for example with a physiotherapist, midwife, a device, or other)? *

- ◯ Yes
- ◯ No

45. Do you perform specific exercises to prevent urinary incontinence? *

- Yes
- No

46. If you experience urine leakage, do you perform specific exercises to control this urinary incontinence? *

- Yes
- No

47. Do you consider breathing during exercise to be important? *

- Yes
- No
- I don't know

48. When lifting weights, during which phase of breathing do you perform the exercise? *

- Inhalation
- Exhalation
- I don't know
- Holding my breath

Questionnaire on Urinary Symptoms (Urinary Symptom Profile - USP)

49. During the past 4 weeks, please specify the number of times you experienced urine leakage during physical exertion: *

Please check one box for each of the lines 49.1, 49.2, and 49.3.

	Never experienced urine leakage	Less than one urine leakage per week	Several urine leakages per week	Several urine leakages per day
49.1 During significant physical exertion	○	○	○	○
49.2. During moderate physical exertion	○	○	○	○
49.3. During light physical exertion	○	○	○	○

During the last four weeks:

(And under the usual conditions of your social, professional or family activities)

50. How many times have you had to rush to the toilet to urinate because of an urgent need? *

- ◯ Never
- ◯ Less than once a week
- ◯ Several times a week
- ◯ Several times a day

51. When you have an urgent need to urinate, how many minutes on average can you hold it in? *

- ◯ More than 15 minutes
- ◯ From 6 to 15 minutes
- ◯ From 1 to 5 minutes
- ◯ Less than 1 minute

52. How often have you experienced urine leakage preceded by an urgent need to urinate that you were unable to control? *

- ◯ Never
- ◯ Less than once a week
- ◯ Several times a week
- ◯ Several times a day

53. In relation to the previous question, in these circumstances, what type of leaks do you have? *

- ◯ No leaks in this circumstance
- ◯ A few drops
- ◯ Small leaks
- ◯ Flooding leaks

54. During the day, how long is the usual interval between two urinations (the act of urinating)? *

- ◯ Two hours or more
- ◯ Between 1 o'clock and 2 o'clock
- ◯ Between 30 minutes and 1 hour
- ◯ Less than 30 minutes

55. How many times on average have you been woken up at night by the need to urinate? *

- ◯ 0 or 1 times
- ◯ twice
- ◯ 3 or 4 times
- ◯ More than 4 times

56. How often have you leaked urine while sleeping or woken up wet? *

- ◯ Never
- ◯ Less than once a week
- ◯ Several times a week
- ◯ Several times a day

57. How would you describe your usual urination (the act of urinating) over the past 4 weeks? *

- ◯ Normal
- ◯ Need to push with abdominal muscles (stomach) or urinate while leaning forward (or requiring a change of position)
- ◯ Need to press down on the lower abdomen with your hands
- ◯ Urinary catheter drainage

58. In general, how would you describe your urine stream? *

- ◯ Normal
- ◯ Low jet
- ◯ Drop by drop
- ◯ Urinary catheter drainage

59. In general, how do you urinate? *

- ◯ Normal and rapid urination
- ◯ Difficulty starting urination, then proceeding normally
- ◯ Miction débutant facilement mais longue à terminer
- ◯ Very slow urination from start to finish
- ◯ Urinary catheter drainage

Este conteúdo não foi criado nem é aprovado pela Microsoft. Os dados que submeter serão enviados para o proprietário do formulário.

Microsoft Forms
